# Addition of Immunosuppressive Treatment to Hemoperfusion Is Associated with Improved Survival after Paraquat Poisoning: A Nationwide Study

**DOI:** 10.1371/journal.pone.0087568

**Published:** 2014-01-27

**Authors:** Wen-Pyng Wu, Ming-Nan Lai, Ching-Heng Lin, Yu-Fen Li, Ching-Yuang Lin, Ming-Ju Wu

**Affiliations:** 1 Graduate Institute of Clinical Medical Science, College of Medicine, China Medical University, Taichung, Taiwan; 2 Division of Nephrology, Chang-Bing Show Chwan Memorial Hospital, Changhua, Taiwan; 3 Department of Statistics, Feng Chia University, Taichung, Taiwan; 4 Department of Medical Research, Taichung Veterans General Hospital, Taichung, Taiwan; 5 Institute of Biostatistics, China Medical University, Taichung, Taiwan; 6 Clinical Immunology Center, China Medical University Hospital, Taichung, Taiwan; 7 Institute of Clinical Medicine, National Yang-Ming University, Taipei, Taiwan; 8 School of Medicine, Chung-Shan Medical University, Taichung, Taiwan; 9 Graduate Institute of Biomedical Science, National Chung Hsing University, Taichung, Taiwan; 10 Division of Nephrology, Department of Medicine, Taichung Veterans General Hospital, Xitun District, Taichung City, Taiwan; Xi'an Jiaotong Univesity School of Medicine, China

## Abstract

Paraquat poisoning associates very high mortality rate. Early treatment with hemoperfusion is strongly suggested by animal and human studies. Although the survival benefit of additional immunosuppressive treatment (IST) in combination with hemoperfusion is also reported since 1971, the large-scale randomized control trials to confirm the effects of IST is difficult to be executed. Therefore, we designed this nationwide large-scale population-based retrospective cohort study to investigate the outcome of paraquat poisoning with hemoperfusion and the additional effects of IST combined with hemoperfusion. This nationwide retrospective cohort study utilized data retrieved from the National Health Insurance Research Database (NHIRD) of Taiwan. A total of 1811 hospitalized patients with a diagnosis of paraquat poisoning who received hemoperfusion between 1997 and 2009 were enrolled. The mean age of all 1811 study subjects was 47.3 years. 70% was male. The overall survival rate was only 26.4%. Respiratory failure and renal failure were diagnosed in 56.2% and 36% patients. The average frequency of hemoperfusion was twice. IST was added in 42.2% patients. IST significantly increases survival rate (from 24.3% to 29.3%, P<0.001). The combined IST with methylprednisolone, cyclophosphamide and dexamethasone associates with the highest survival rate (48%, P<0.001). Moreover, patients younger than 45 years of age in the IST group had the best survival (41.0% vs. 33.7%, p<0.001). Our results support the use of IST with hemoperfusion for paraquat-poisoned patients. The best survival effect of IST is the combination of methylprednisolone, cyclophosphamide and daily dexamethasone, especially in patients with younger age.

## Introduction

Paraquat is a highly toxic herbicide that is used worldwide. Paraquat poisoning remains a major cause of death among patients with acute poisoning in developing countries [1]. Paraquat was first described by Weidel and Rosso in 1882 [2]. Generally, management of victims of paraquat poisoning includes activated charcoal administered via a nasogastric tube to prevent absorption by the gastrointestinal tract [3], [4]. In order to increase the elimination of paraquat from plasma, the physicians often choose activated charcoal hemoperfusion therapy (6–8 hours) for 2 or more sessions [2], [5]–[7]. Beyond the positive results from canine model of paraquat poisoning, Hsu and colleague reported that early hemoperfusion improves survival of severe paraquat-poisoned patients [8]–[10] Radiotherapy, desferoxamine, immunosuppressive treatment (IST), or vitamin E have been used with various success in paraquat poisoning, but the evidence is not particularly strong [11]–[15].

In order to reduce the extent of pulmonary inflammation and fibrosis induced by paraquat, anti-inflammatory and immunosuppressive agents such as cyclophosphamide (CP) and steroids have been applied. IST in combination with hemoperfusion for paraquat poisoning was first reported by Malone et al. in 1971 [16], and further reports were published thereafter [17]–[22]. According to a review of recent studies, inflammation appears to develop as an early response of the lung to paraquat poisoning [2], [23]–[25]. The inflammatory cells generate reactive oxygen species (ROS) [2], [25] and proteolytic enzymes (such as elastase) [26], leading to the development of pulmonary inflammation and fibrosis.

In 1984, Addo and Poon-King reported a combination of high-dosage CP (5 mg/kg/day, i.v.) and dexamethasone (DEX, 24 mg/day i.v.) treatment for 14 days yielded a survival rate of 75% in patients with paraquat poisoning [5], [27]. However, Perriens and colleagues reported no survival benefit using the same treatment protocol in 1992 [5], [28]. In 1996, Lin et al. [17] used pulse therapy with CP (1 g/day) for two days and MP (1 g/day) for three days in patients with moderate to severe paraquat poisoning because of encouraging reports of its use in patients with severe lung damage due to systemic lupus erythematosus (SLE) [29], [30] and Wegener's granulomatosis [31]. The results revealed mortality in the pulse therapy group was lower than that in the control group (4 of 16, 25%, vs. 12 of 17, 70.6%, p = 0.01) [17]. In 2008, Afzali and Gholyaf also reported that CP and MP pulse therapy may be effective in preventing respiratory failure and reducing mortality in patients with moderate to severe paraquat poisoning [32].

Because the number of cases of paraquat poisoning in a single center is usually small, it is difficult to conduct a large-scale randomized control trial to compare the effects of various ISTs in combination with hemoperfusion. Studies on treatment effects in paraquat poisoning must comply with ethical guidelines and therefore the use of control groups may not be permitted due to potentially high mortality rates. Furthermore, the numbers of cases of paraquat poisoning in the literature were small, and the use of IST for paraquat poisoning is thus still considered an experimental therapy [2]. Therefore, we designed a large-scale nationwide population-based retrospective cohort study from the National Health Insurance Research Database (NHIRD) of Taiwan to evaluate the clinical outcome of paraquat-poisoned patients with hemoperfusion treatment and the survival benefit of additional IST in combination with hemoperfusion treatment.

## Materials and Methods

### Ethics statement

This study was approved by the National Health Research Institutes of Taiwan. Because the identification numbers and personal information of the individuals included in the study were not included in the secondary files, the review board of the Institutional Review Board of Taichung Veterans General Hospital stated that written consent from patients was not required.

### Study population and data collection

This study was designed as a nationwide population-based retrospective cohort study. All data were obtained from the NHIRD of Taiwan. The NHIRD has been described in detail in previous studies [33], [34]. In brief, Taiwan initiated a single-payer National Health Insurance program on March 1, 1995. In 2009, the database consisted of health care data from more than 99% of the entire population of 23.12 million. The NHIRD contains registration files and original claims data for reimbursement. The databases derived from this system by the Bureau of National Health Insurance, Taiwan (BNHI) and maintained by the National Health Research Institutes, Taiwan, are provided to scientists in Taiwan for research purposes. The International Classification of Diseases, Ninth Revision, Clinical Modification (ICD-9-CM) codes were used to define diseases.

**Figure 1:**
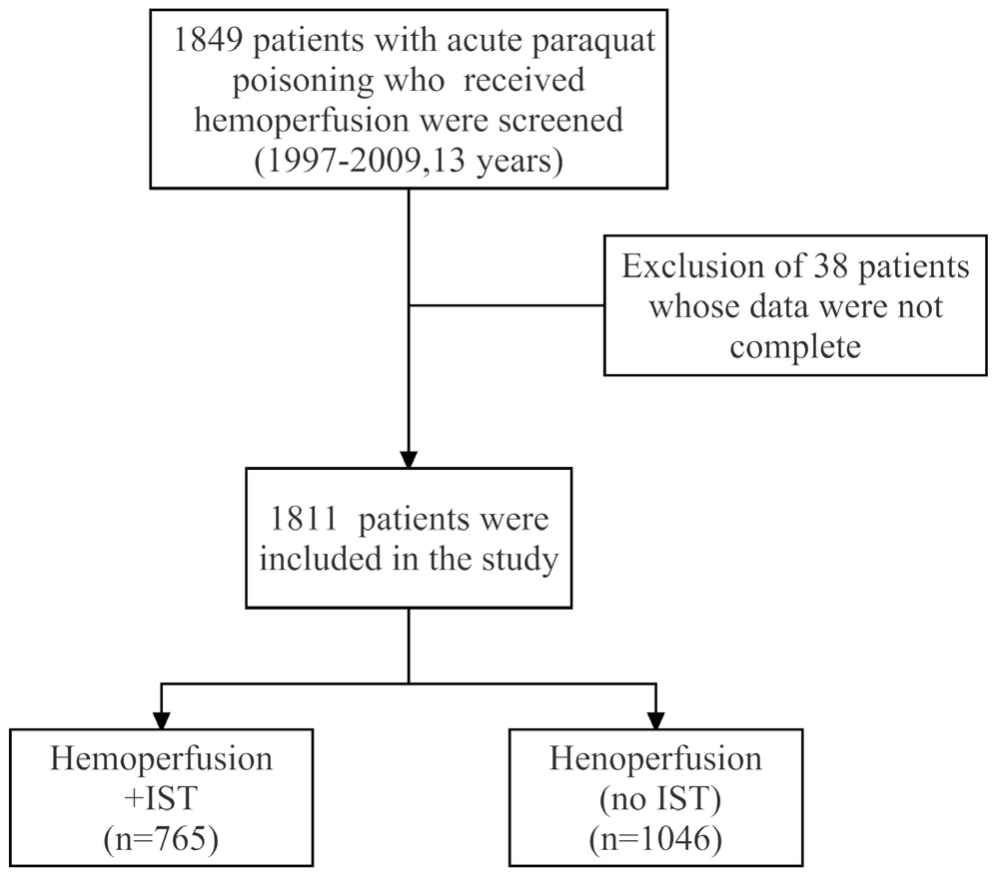
Selection and grouping of the patients with paraquat poisoning. Abbreviations: IST: Immunosuppressive treatment.

From this database, we selected patients who were admitted to a hospital between January 1, 1997 and December 31, 2009, with a primary diagnosis of paraquat poisoning and treated with hemoperfusion (ICD-9 codes: 989.4 plus treatment code for hemoperfusion, with or without E-codes E863.5, E980.7, E950.6 or E962.1). In this 13-year period, 2830 patients received hemoperfusion. Of these patients, 1849 patients were diagnosed with paraquat poisoning and received hemoperfusion. After we excluded 38 patients with incomplete data, the remaining 1811 patients were included in the study for further analysis (Fig. 1).

The NHIRD contains details of every prescription including dose, frequency, and starting date of the drug administration. In the database, we could not evaluate the quantity of paraquat ingested, time from ingestion to evaluation to medical care or time from ingestion to treatment. All of the information about medications for individual patients was obtained from the NHIRD. Those patients who received MP, CP or daily DEX therapy were assigned to the IST group (n = 765). MP pulse therapy was defined as a dose of MP greater than 500 mg and CP pulse therapy was defined as a dose of CP greater than 200 mg. The non-IST group consisted of patients who received hemoperfusion alone (n = 1046).

The IST group was further divided into 7 groups, as follows: MP pulse, CP pulse, daily DEX therapy, MP+CP, MP+DEX, CP+DEX and MP+CP+DEX group. The hazard ratio (HR) of each group versus the HR in the non-IST group was evaluated. The definition of mortality is mainly according to the discharge condition. If the discharge status is death or discharge against medical advice (DAMA) in critical condition, we would also check the insurance status. It these patients also quit health insurance after discharge and did not have any medical record after discharge, we defined these patients as mortality cases.

### Statistical analysis

Independent t-test and Chi-square test were used for analysis of the baseline data of each group. Kaplan-Meier survival plots were compared using log-rank tests to assess the relationships between the treatment protocols and mortality. A Cox proportional hazards regression model was applied to determine the significance of the baseline variables for predicting death from paraquat poisoning. The hazard ratios for mortality and 95% confidence intervals (CIs) were calculated. All of the above analyses were conducted using SAS (version 9.3; SAS Institute, Inc., Cary, NC) and SPSS statistical software (version 12.0; SPSS, Inc., Chicago, IL). All statistical tests were two-sided and statistical significance was defined as a p-value <0.05.

## Results

Totally, 1811 hospitalized paraquat-poisoned patients with hemoperfusion treatment from 64 hospitals between 1997 and 2009 were enrolled. The mean age was 47.3±17.6 years and ranged between 3 and 93 years, 1268 (70%) patients were male. The overall survival rate was only 26.4%. The average frequency of hemoperfusion was 2.0±1.4 and the average hospitalization days was 6.5±11.0. Of them, 1018 (56.2%) patients developed respiratory failure and 861 (47.5%) received ventilator support; while 651 (36%) patients had acute kidney injury and 232 (12.8%) received emergent hemodialysis. IST was used in 765 (42.2%) patients. The percentage of the use of MP pulse, CP pulse and continuous daily DEX in paraquat-poisoned patients were 31.4%, 23.7% and 16.3%, respectively (Table 1). The percentages of patients treated in medical centers, regional hospitals or district hospitals were 42.13% (n = 763), 50.64% (n = 917) and 7.23% (n = 131), respectively.

**Table 1:** Characteristics of study subjects classified by survival outcome.

	Total		Survival		Mortality		P
	(n = 1811)		(n = 479)		(n = 1332)		(Survival vs. Mortality)
Age, years (mean±SD)	47.3	±17.6	38.6	±14.9	50.4	±17.5	<0.001
Male gender, n (%)	1268	(70.0%)	313	(65.3%)	955	(71.7%)	0.009
Hospitalization days (mean±SD)	6.5	±11.0	13.4	±16.7	4.1	±6.5	<0.001
Frequency of HP (mean±SD)	2.0	±1.4	2.4	±1.6	1.8	±1.3	<0.001
RF, n (%)	1018	(56.2%)	68	(14.2%)	950	(71.3%)	<0.001
RF with ventilator support, n (%)	861	(47.5%)	58	(12.1%)	803	(60.3%)	<0.001
RF without ventilator support, n (%)	157	(8.7%)	10	(2.1%)	147	(11.0%)	<0.001
AKI, n (%)	651	(36.0%)	132	(27.6%)	519	(39.0%)	<0.001
AKI with HD, n (%)	232	(12.8%)	33	(6.9%)	199	(15.0%)	<0.001
AKI without HD, n (%)	419	(23.1%)	99	(20.7%)	320	(24.0%)	0.135
IST, n (%)	765	(42.2%)	225	(47.0%)	540	(40.5%)	0.015
MP pulse, n (%)	568	(31.4%)	162	(33.8%)	406	(30.5%)	0.177
CP pulse, n (%)	429	(23.7%)	131	(27.3%)	298	(22.4%)	0.028
DEX, n (%)	295	(16.3%)	115	(24.0%)	180	(13.5%)	<0.001
Comorbidity							
DM, n (%)	185	(10.2%)	32	(6.7%)	153	(11.5%)	0.003
HTN, n (%)	306	(17.9%)	53	(11.7%)	253	(19.0%)	<0.001
CVA, n (%)	72	(4.0%)	5	(1.0%)	67	(5.0%)	<0.001
CHF, n (%)	103	(5.7%)	10	(2.1%)	93	(7.0%)	<0.001
CAD, n (%)	103	(5.7%)	15	(3.1%)	88	(6.6%)	0.005
COPD, n (%)	30	(1.88%)	1	(0.2%)	29	(2.2%)	0.004
Cancer, n (%)	107	(5.9%)	18	(3.8%)	89	(6.7%)	0.020

Abbreviations: HP: Hemoperfusion; RF: Respiratory failure; AKI: Acute kidney injury; IST: Immunosuppressive treatment; MP: Methylprednisolone; CP: Cyclophosphamide; DEX: Dexamethasone; DM: Diabetes mellitus; HTN: Hypertension; CVA: Cerebral vascular accident; CHF: Congestive heart failure; CAD: Coronary artery disease; COPD: Chronic obstructive pulmonary disease.

* Independent t-test and Chi-square test were used for analysis.

### Differences between survival and mortality groups

The analysis of differences between the survival and mortality groups revealed younger age, greater number of hospitalization days and higher frequency of hemoperfusion in the survival group (Table 1). In addition, a lower percentage of males, lower ventilator use and lower hemodialysis rate for acute kidney injury (all P<0.01) were found in the survival group. The percentage of patients who received IST was also higher (47.0% vs. 40.5%, P = 0.015) in the survival group. All ISTs, including MP pulse, CP pulse and DEX, were prescribed more frequently in the survival group than in the mortality group.

### The IST group had better survival than the non-IST group

**Figure 2:**
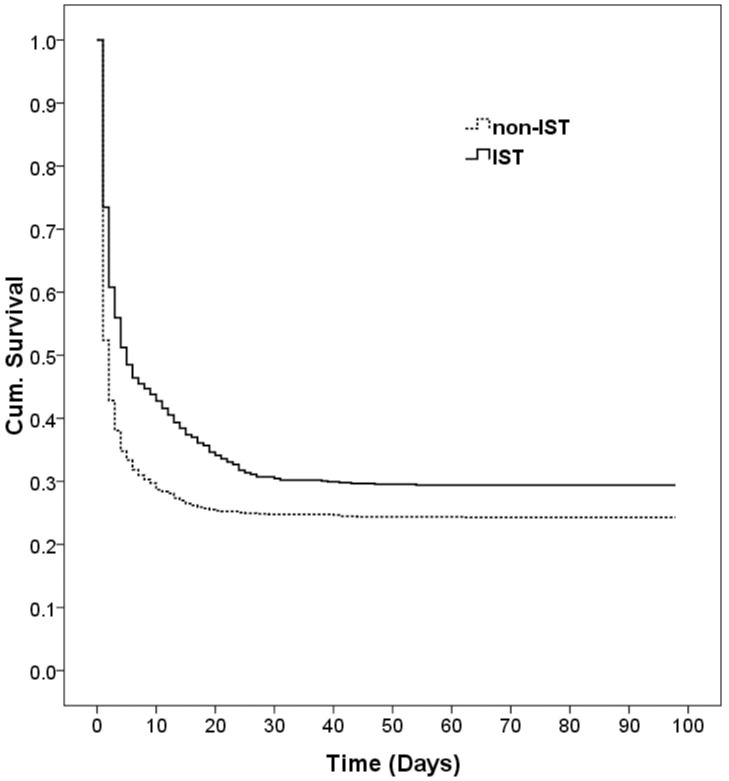
Kaplan-Meier survival plot of the IST group (n = 765) versus the non-IST control group (n = 1046, log-rank test, p< = 0.001).

The IST and non-IST groups consisted of 765 (42.2%) and 1046 (57.8%) patients, respectively. The baseline data of IST and non-IST groups are listed in Table 2. There were no significant differences between the two groups in gender, but there were significant differences in age (45.8±17.4 vs. 48.3±17.8, p = 0.003) and length of hospital stay (8.9±14.7 vs. 4.8±6.7 days, p<0.001). There were also no significant differences in comorbidities between IST and non-IST groups except in cancer (7.2% vs.5.0%, p = 0.048, Table 2). A significant increase of survival rate was found in the IST group (29.3%, 225/765) compared to non-IST group (24.3%, 257/1046; log-rank test, P<0.001; Fig. 2).

**Table 2:** Characteristics of study subjects with or without immunosuppressive treatment.

	IST		Non-IST		Significance	
	(n = 765)		(n = 1046)		P	Hazard ratio (95% CI)
HP use, n (%)	765	(100.0%)	1046	(100.0%)		
Survival cases, n (%)	225	(29.3%)	254	(24.3%)	<0.001	0.825# (0.739–0.921)
Male gender, n (%)	542	(70.8%)	726	(69.4%)	0.542	
Age, years (mean±SD)	45.8	±17.4	48.3	±17.8	0.003	
Length of Hospital Stay, days (mean±SD)	8.9	±14.7	4.8	±6.7	<0.001	
Comorbidity						
ESRD, n (%)	3	(0.4%)	3	(0.3%)	0.700	
DM, n (%)	79	(10.3%)	106	(10.1%)	0.894	
HTN, n (%)	120	(15.7%)	186	(17.8%)	0.240	
CVA, n (%)	29	(3.8%)	43	(4.1%)	0.731	
CHF, n (%)	42	(5.5%)	61	(5.8%)	0.757	
CAD, n (%)	45	(5.9%)	58	(5.5%)	0.759	
COPD, n (%)	13	(1.7%)	17	(1.6%)	0.902	
Cancer, n (%)	55	(7.2%)	52	(5.0%)	0.048	

Abbreviations: IST: Immunosuppressive treatment; HP: Hemoperfusion; ESRD: End stage renal disease; DM: Diabetes mellitus; HTN: Hypertension; CVA: Cerebral vascular accident; CHF: Congestive heart failure; CAD: Coronary artery disease; COPD: Chronic obstructive pulmonary disease.

* Independent t-test, Chi-square test and Cox regression analysis were used for analysis.

# Cox regression analysis among different treatment groups, adjusted for age and sex.

We further check the crude or adjusted (age and sex) hazard ratios of the comorbidities by Cox regression analysis. The adjusted hazard ratio for respiratory failure was 2.721 (95% CI: 2.406–3.078, P<0.001, Table 3), but the adjusted hazard ratio for acute kidney injury was only 1.092 (95% CI: 0.977–1.220, P =  0.121, Table 3). Other comorbidities including diabetes mellitus (DM), hypertension (HTN), cerebral vascular accident (CVA), congestive heart failure (CHD), coronary artery disease (CAD), chronic obstructive pulmonary disease (COPD) and cancers all had higher hazard ratio (1.305–2.002, Table 3) if not adjusted by age and sex. Interestingly, it was only significant in CHF if the hazard ratio was adjusted by age and sex.

**Table 3:** Hazard ratio and adjusted (age and sex) hazard ratio in patients with paraquat poisoning with different comorbidities.

	No.	Survival	HR	95% CI	P value	Adjusted HR	95% CI	P value
RF, n (%)	1018	6.7%	2.938	2.600–3.319	<0.001	2.721	2.406–3.078	<0.001
RF with ventilator support	861	6.7%	2.383	2.130–2.667	<0.001	2.217	1.980–2.483	<0.001
RF without ventilator support	157	6.4%	1.529	1.287–1.816	<0.001	1.466	1.234–1.742	<0.001
AKI, n (%)	651	20.3%	1.074	0.961–1.199	0.207	1.092	0.977–1.220	0.121
AKI with HD	232	14.2%	1.029	0.884–1.196	0.714	1.019	0.876–1.186	0.807
AKI without HD	419	23.6%	1.077	0.949–1.221	0.250	1.107	0.976–1.256	0.113
DM	185	17.3%	1.349	1.140–1.597	<0.001	1.035	0.870–.231	0.698
HTN	306	17.3%	1.428	1.245–1.639	<0.001	1.051	0.908–1.218	0.502
CVA	72	6.9%	1.754	1.370–2.244	<0.001	1.262	0.981–1.624	0.071
CHF	103	9.7%	1.498	1.212–1.850	<0.001	1.271	1.027–1.572	0.027
CAD	103	14.6%	1.489	1.199–1.849	<0.001	1.020	0.814–1.280	0.861
COPD	30	3.3%	2.002	1.384–2.895	<0.001	1.302	0.894–1.896	0.169
Cancer	107	16.8%	1.305	1.052–1.619	0.015	1.059	0.851–1.317	0.609

Abbreviations: HR: Hazard ratio; RF: Respiratory failure; AKI: Acute kidney injury; HD: Hemodialysis; DM: Diabetes mellitus; HTN: Hypertension; CVA: Cerebral vascular accident; CHF: Congestive heart failure; CAD: Coronary artery disease; COPD: Chronic obstructive pulmonary disease.

* Cox regression analysis among different treatment groups adjusted for age and sex or not adjusted were used for analysis.

### The best treatment regimen for paraquat-poisoned patients with hemoperfusion treatment was the three-combined treatment of MP, CP and daily DEX

**Figure 3:**
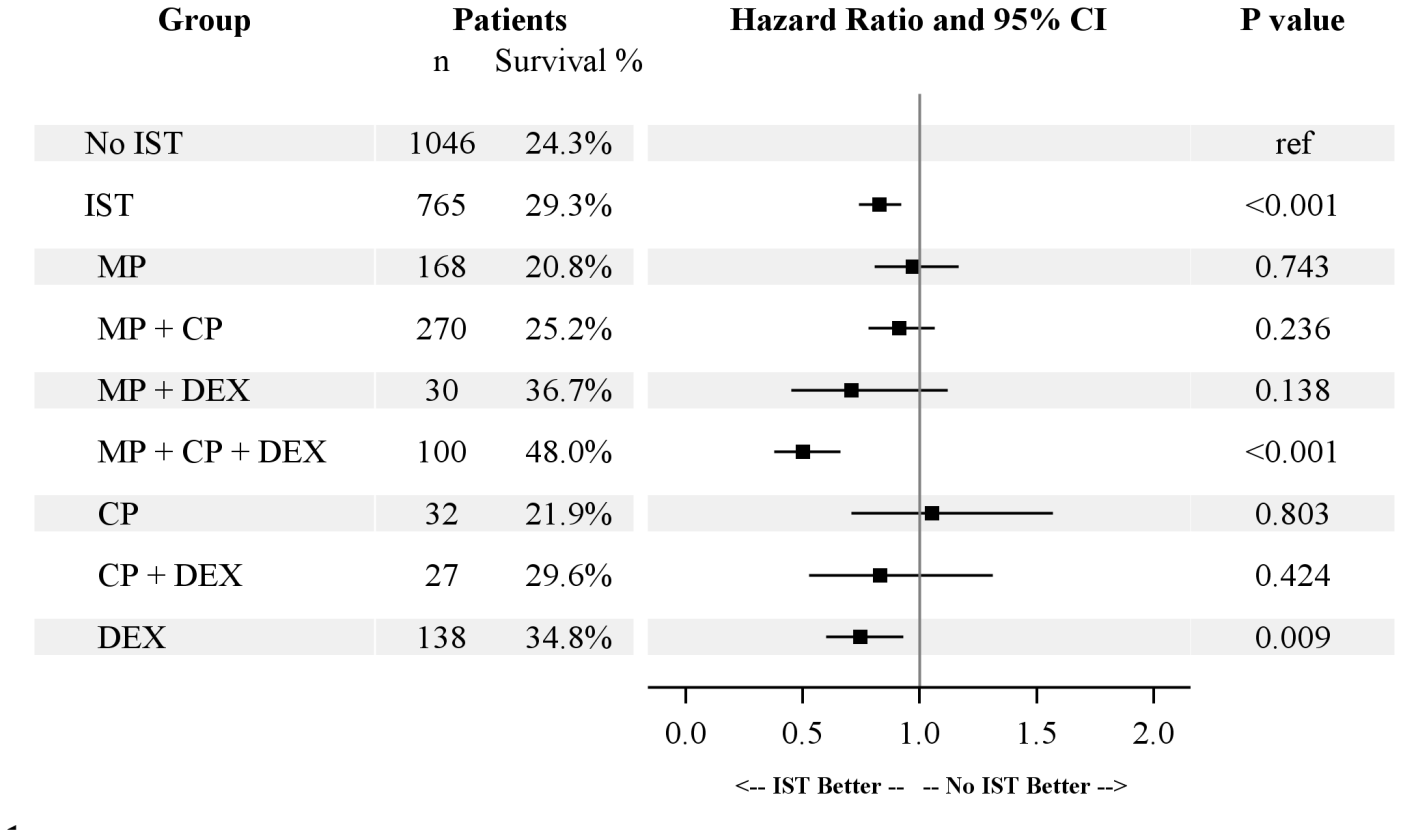
Treatment effects of different immunosuppressive regimens in paraquat-poisoned patients with hemoperfusion treatment. Cox regression analysis of mortality rates among different treatment groups, adjusted for age and sex, was used to calculate the hazard ratio. Abbreviations: No IST: No immunosuppressive treatment (Hemoperfusion alone); IST: Immunosuppressive treatment; MP: Methylprednisolone; CP: Cyclophosphamide; DEX: Dexamethasone.

In the IST group, the mean MP dose was 1733.1±1014.3 (40–13500) mg, the mean CP dose was 2532.1±1629.7 (40–13500) mg, and the mean DEX daily dose was 14.4±14.1 (0.2–100.0) mg/day. We further divided the IST group into seven subgroups: MP, CP, DEX, MP+CP+DEX, MP+CP, MP+DEX, and CP+DEX. The patient number of each subgroup was listed in Figure 3.

**Figure 4:**
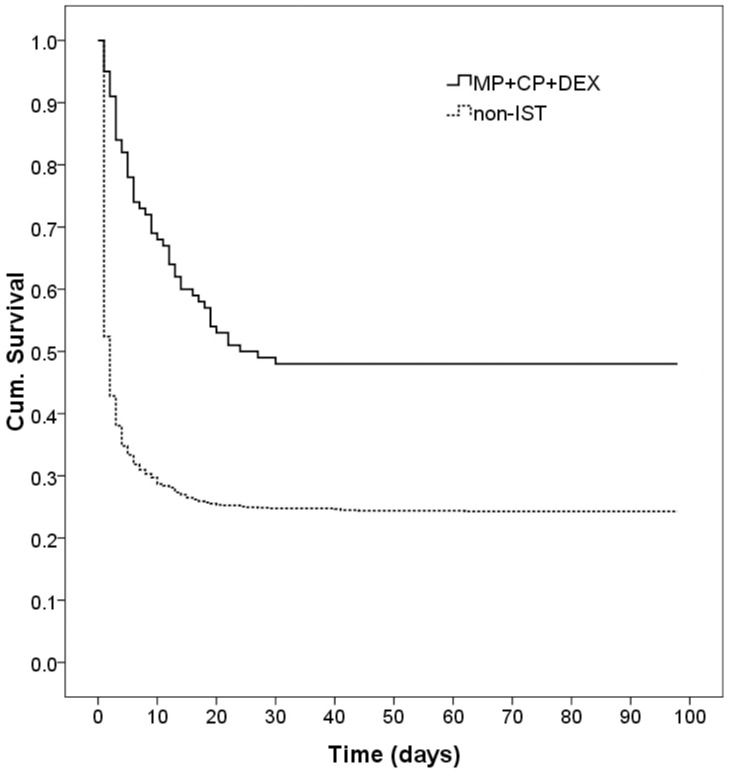
Kaplan-Meier survival plot of the MP+CP+DEX subgroup (n = 100) versus the non-IST group (n = 1046, log rank test, P<0.001). Abbreviations: MP: Methylprednisolone; CP: Cyclophosphamide; DEX: Dexamethasone; IST: Immunosuppressive treatment.

The MP+CP+DEX subgroup had the highest survival rate (48.0%, Fig. 3), followed by the MP+DEX subgroup (36.7%), DEX subgroup (34.8%), CP+DEX subgroup (29.6%), MP+CP subgroup (25.2%), CP subgroup (21.9%) and MP subgroup (20.8%). When compared with the non-IST group by Cox regression adjusted for age and sex, the hazard ratio (HR) of the MP+CP+DEX subgroup was 0.499 (95% CI: 0.376–0.661, P<0.001, Fig. 3) and the hazard ratio of the DEX subgroup was 0.746 (95% CI: 0.599–0.928, P = 0.009). The Kaplan-Meier survival analysis also revealed a significantly higher survival rate in the MP+CP+DEX subgroup (48.0%, 48/100) than in the non-IST group (24.3%, 257/1046; log-rank test, P<0.001; Fig. 4). The hazard ratios of the MP+DEX (HR: 0.708, 95% CI: 0.449–1.118, P = 0.138) and CP+DEX subgroups (HR: 0.830, 95% CI: 0.526–1.310, P = 0.424) were also lower but they were not significant. A possible explanation for this finding may be that there were fewer cases in these two subgroups (n = 30 and n = 27, respectively). The hazard ratio of the MP+CP subgroup (HR: 0.911, 95% CI: 0.780–1.063, P = 0.236) was also less than 1, but this was not significant. The MP (HR: 0.970, 95% CI: 0.807–1.166, P = 0.743) and CP subgroups (HR: 1.052, 95% CI: 0.706–1.567, P = 0.803) showed no significant differences compared with the non-IST group.

**Figure 5:**
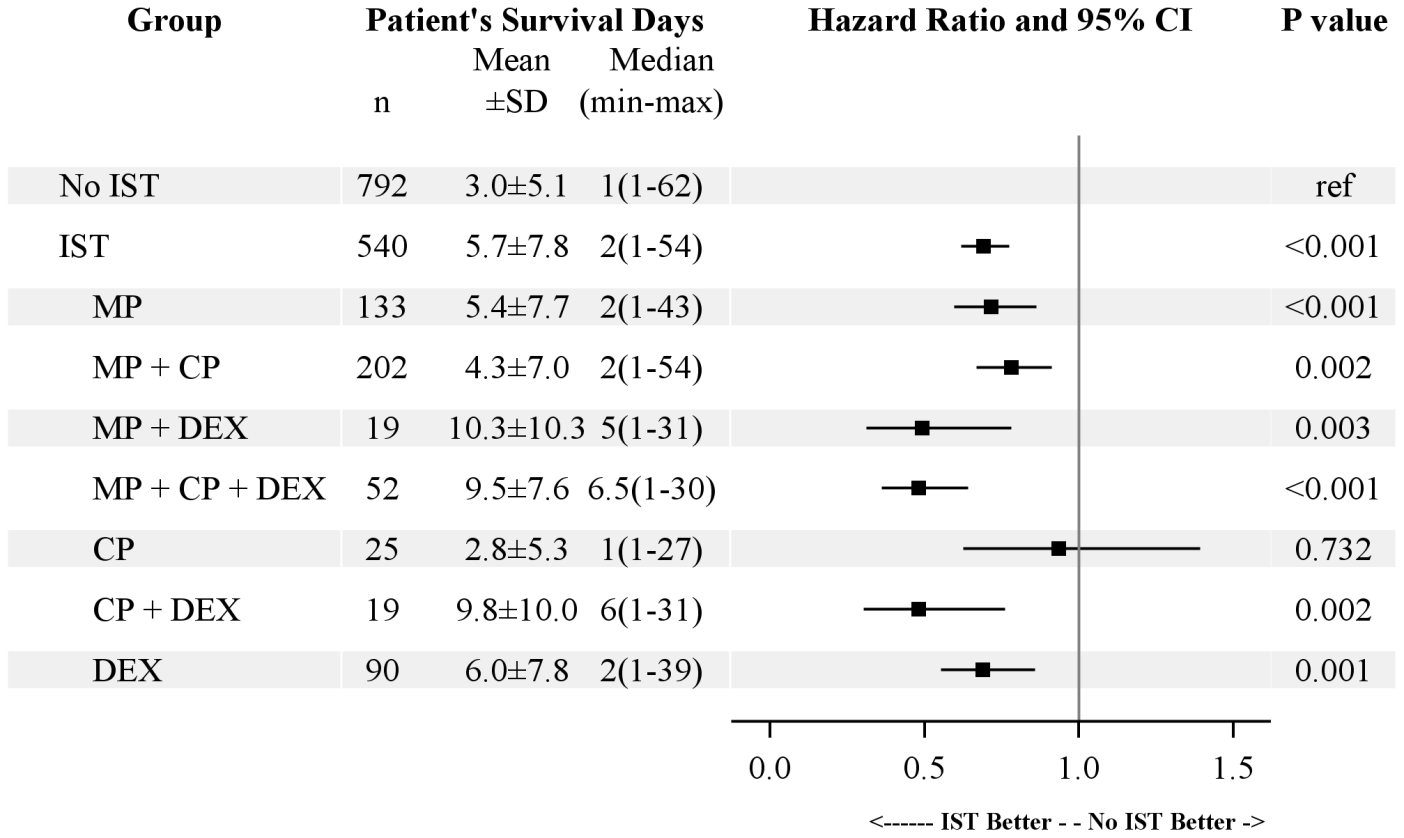
Comparison of survival day before mortality in paraquat-poisoned patients with additional different combined immunosuppressive regimens and hemoperfusion. Cox regression analysis of mortality rates among different treatment groups, adjusted for age and sex, was used to calculate the hazard ratio. All patients received hemoperfusion. Abbreviations: No IST: No immunosuppressive treatment (Hemoperfusion alone); IST: Immunosuppressive treatment; MP: Methylprednisolone; CP: Cyclophosphamide; DEX: Dexamethasone.

In order to evaluate whether IST delayed mortality, we analyzed the number of survival days between patients who died in the IST and non-IST groups after paraquat poisoning. The survival duration of IST group was longer than the non-IST group (5.7±7.8 vs. 3.0±5.1 days, p<0.001, Fig. 5). The analysis of treatment subgroups also revealed significantly longer survival in all subgroups, except the CP pulse subgroup. This finding implies that the treatment regimens of MP pulse and MP+CP pulse may prolong the number of survival days, even though the survival rates were not higher than that of the non-IST group.

We further presumed that a CP dose greater than 3 g or an MP dose greater than 4 g was possible in cases who received repeat CP or MP pulse therapy. The survival rate of patients with repeat pulse therapy was 33.9% (42/124; HR: 0.653, 95% CI: 0.519–0.822), and the p-value was 0.0003 when compared with the non-IST group (24.3%, 254/1046) by Cox regression adjusted for age and sex. The survival rate of patients with repeat pulse therapy in the MP+CP+DEX subgroup was 30.0% (12/40, HR: 0.670, 95% CI: 0.459–0.979), and the p value was 0.038 when compared with the non-IST group by Cox regression adjusted for age and sex.

### Patients younger than 45 years of age responded best to the immunosuppressive treatment

A comparison of the survival and mortality groups showed that mean age was lower in the survival group (38.6±14.9 vs. 50.4±17.5, p<0.001, Table 1). Because age was an important factor for survival, we further divided the patients into three subgroups by age. The survival was better in the IST group only among patients who were younger than 45 years of age (41.0% vs. 33.7%, HR: 0.734, p<0.001). There was no difference in survival benefit of IST in the groups whose ages were between 45 and 64 (20.6% vs. 20.1%, HR: 0.874, p = 0.181) or over 65 (7.4% vs.10.0%, HR: 0.935, p = 0.550).

## Discussion

The present study is, to the best of the authors' knowledge, the largest study to date to evaluate the benefit of IST in combination with hemoperfusion for paraquat poisoning. We found a very high mortality rate, 73.6%, in paraquat-poisoned patients. The paraquat-poisoned patients with hemoperfusion treatment have additional survival benefit from IST. The patients who received the three-combined therapy of MP and CP with daily DEX had the best survival benefit (HR: 0.5, P<0.001). This finding provides clinical evidence for the beneficial effect of IST in paraquat poisoning.

We chose the ICD-9 code “989.4”, which is coding for paraquat poisoning [35] and represented “toxic effect of other pesticides, not elsewhere classified”, with hemoperfusion for paraquat poisoning, because the diagnosis included in the ICD-9 number which is indicated for hemoperfusion in Taiwan is paraquat poisoning.

The major ISTs prescribed for paraquat poisoning include DEX, CP and MP [19], [27], [36]-[38]. These regimens all resulted in good survival rates in several studies but due to the small sample sizes, bias may have existed [22]. In order to include a larger number of patients in our analysis, we selected patients from Taiwan's National Health Insurance Research Database (NHIRD) from 1997 to 2009 to evaluate the survival benefit of IST for patients with paraquat poisoning. A total of 1811 cases from 64 different hospitals were included in the final analysis in this study. We selected paraquat-poisoned patients who received a therapy of MP, CP or DEX, or a combination thereof, to be included in the IST group. The IST group had significantly lower mortality rate than the non-IST group. We further divided the IST group into 7 subgroups, i.e., patients who used the three aforementioned drugs separately or in different combinations. The combined treatment protocol of MP and CP with daily DEX had the best survival benefit. In contrast, the survival rate of patients who received MP alone was the lowest. Although the MP and CP subgroups did not have significantly higher survival rates, they showed a significantly higher number of survival days compared with that of the non-IST group among patients who subsequently died of paraquat poisoning. Therefore, MP and CP pulse therapy showed a treatment benefit in paraquat poisoning. We hypothesize that the initial MP and CP pulse therapies are important for rapid suppression of the paraquat-induced inflammation. Daily DEX is also necessary for continuous suppression of inflammation to reduce the toxic effect of paraquat and prevent lethal pulmonary inflammation.

Lin et al. reported improved survival in severe paraquat poisoning with repeated MP and CP pulse therapy combined with continuous DEX therapy in 2006 and 2011 [19], [38]. The indication for repeated MP and CP pulse therapies was a PaO2 level lower than 60 mmHg in both of their studies. In our study, the patients who received repeat CP and MP pulse therapy had a higher survival rate than non-IST patients, but there was no difference in survival compared with patients in the MP+CP+DEX group. This may be due to the greater severity of paraquat poisoning in patients who needed repeat pulse therapy compared with patients in the MP+CP+DEX group.

Our finding that elderly persons with paraquat poisoning had a higher mortality rate supports the results of previous studies [38]–[40]. We further found that patients younger than 45 years who received IST showed the best survival. Possible reasons may be better tolerance of IST and lower co-morbidity in younger patients. Thus, we strongly recommend the use of IST, especially the three-combined regimen of MP+CP+DEX, in paraquat-poisoned patients younger than 45 years. Moreover, similar to the treatment of hemoperfusion, the use of IST should be as early as possible. IST could be used earlier than hemoperfusion. Further study to compare the timing of IST is necessary to confirm this concept.

There were some limitations in the study. First, it was not a randomized controlled clinical trial. Because cases of paraquat poisoning are rare and there are ethical considerations, it is very difficult to conduct a large randomized controlled trial. This nationwide population-based retrospective cohort study could be a better approach to overcome the problems of small sample size and the use of data from a single center, as seen in previous studies [17], [19], [37], [38]. Second, we defined patients who received hemoperfusion and ICD-9 coding for paraquat poisoning as cases of paraquat poisoning. While the possibility of patients having other diseases could not be absolutely excluded, exceptions are likely to be extremely rare and therefore their confounding influence in the present analysis was probably negligible. Third, there were no data regarding blood or urine levels of paraquat in the database of NHIRD. The record of duration between paraquat intoxication and treatment was also not available in this database. Therefore, the evaluation of disease activity was difficult. So this study aimed on the additional effect of IST in paraquat-poisoned patients who have already received hemoperfusion.

## Conclusions

In summary, our analysis of a large number of cases from multiple centers provides another evidence of a survival benefit conferred by IST in paraquat-poisoned patients. Additional IST with MP, CP and continuous DEX significantly improves outcome in paraquat-poisoned patients with hemoperfusion, especially in patients younger than 45 years.
